# Toxicity of Glycyl-l-Prolyl-l-Glutamate Pseudotripeptides: Cytotoxic, Oxidative, Genotoxic, and Embryotoxic Perspectives

**DOI:** 10.1155/2022/3775194

**Published:** 2022-11-19

**Authors:** Hasan Turkez, Ozlem Ozdemir Tozlu, Arzu Tatar, Mehmet Enes Arslan, Kenan Cadirci, Lisa Marinelli, Omer Erkan Yapca, Ivana Cacciatore, Antonio Di Stefano, Adil Mardinoglu

**Affiliations:** ^1^Department of Medical Biology, Faculty of Medicine, Atatürk University, Erzurum, Turkey; ^2^Erzurum Technical University, Faculty of Science, Department of Molecular Biology and Genetics, Erzurum, Turkey; ^3^Department of Otorhinolaryngology, Faculty of Medicine, Atatürk University, Erzurum, Turkey; ^4^Department of Internal Medicine, Erzurum Regional Training and Research Hospital, Health Sciences University, Erzurum, Turkey; ^5^Department of Pharmacy, University “G. D'Annunzio” of Chieti-Pescara, Chieti Scalo, Chieti, Italy; ^6^Department of Obstetrics and Gynecology, Faculty of Medicine, Atatürk University, Erzurum, Turkey; ^7^Science for Life Laboratory, KTH-Royal Institute of Technology, Stockholm, Sweden; ^8^Centre for Host-Microbiome Interactions, Dental Institute, King's College London, London SE1 9RT, UK

## Abstract

The tripeptide H-Gly-Pro-Glu-OH (GPE) and its analogs began to take much interest from scientists for developing effective novel molecules in the treatment of several disorders including Alzheimer's disease, Parkinson's disease, and stroke. The peptidomimetics of GPEs exerted significant biological properties involving anti-inflammatory, antiapoptotic, and anticancer properties. The assessments of their hematological toxicity potentials are critically required for their possible usage in further preclinical and clinical trials against a wide range of pathological conditions. However, there is so limited information on the safety profiling of GPE and its analogs on human blood tissue from cytotoxic, oxidative, and genotoxic perspectives. And, their embryotoxicity potentials were not investigated yet. Therefore, in this study, measurements of mitochondrial viability (using MTT assay) and lactate dehydrogenase (LDH) release as well as total antioxidant capacity (TAC) assays were performed on cultured human whole blood cells after treatment with GPE and its three novel peptidomimetics for 72 h. Sister chromatid exchange (SCE), micronucleus (MN), and 8-oxo-2-deoxyguanosine (8-OH-dG) assays were performed for determining the genotoxic damage potentials. In addition, the nuclear division index (NDI) was figured out for revealing their cytostatic potentials. Embryotoxicity assessments were performed on cultured human pluripotent NT2 embryonal carcinoma cells by MTT and LDH assays. The present results from cytotoxicity, oxidative, genotoxicity, and embryotoxicity testing clearly propounded that GPEs had good biosafety profiles and were trouble-free from the toxicological point of view. Noncytotoxic, antioxidative, nongenotoxic, noncytostatic, and nonembryotoxic features of GPE analogs are worthwhile exploring further and may exert high potentials for improving the development of novel disease-modifying agents.

## 1. Introduction

**Figure 1:**
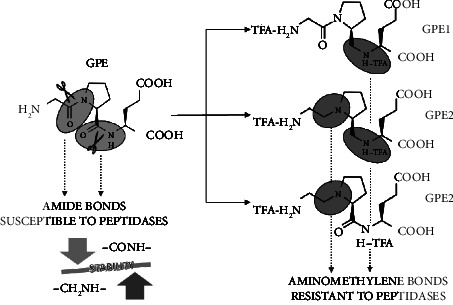
The examined GPEs peptidomimetics synthesized from natural GPE.

The small peptide glycine-L-proline-L-glutamate (GPE) (Figure 1) is naturally cleaved from the N-terminal sequence of insulin-like growth factor (IGF-1) by proteases. There is a commercially available form of this tripeptide known as Glypromate [1, 2]. GPE and its analogs exert key biological functions. Principally GPE and its modified peptidomimetics exhibit neuro-modulatory and neuroprotective properties by providing protection against toxic insults by N-methyl-D-aspartate (NMDA) and amyloid beta, giving countenance to the release of neurotransmitters as well as reducing inflammatory environment and activating different pathways involved in prosurvival [3–5]. In addition to these actions antioxidative, proliferative, and antiapoptotic features by GPE analogs were also previously documented [6–10].

The usage of GPE as a therapeutic tripeptide is limited due to its certain biochemical and pharmacokinetic natures. In that, the presence of degradation process by peptidases and metabolic stability issues are considered as the main limitedness. Correspondingly, GPE exhibited poor delivery to the central nervous system (CNS) due to a very short plasma half-life time (*t*1/2 < 1 h). Hence, it seems to be very critical to develop effective modifying strategies towards neuropeptides involving GPE for dissipating available limitations. In fact, different strategies are being introduced for modifying GPE tripeptide [11–13]. In a recent effort, the amino acids modification of the GPE sequence was practiced for the purpose of enhancing its resistance against proteases and still maintaining its cytoprotective action. Ultimately, three different novels of GPE peptidomimetics shortened as GPE1, GPE2, and GPE3 were synthesized via the reducement of the peptidic bond to an aminomethylenic group at the Pro-Glu (GPE1), Gly-Pro (GPE3), or both the junctions (GPE2) (Figure 1). These modified GPEs exhibited longer plasma half-life time (*t*1/2 > 4.5 h) [5].

Recently small peptides such as GPEs have attracted extensive attention as multipotent therapeutics for the treatment of several diseases including Alzheimer's and Parkinson's diseases, stroke, diabetes as well as some types of cancer [10, 14, 15]. Although the peptidomimetics field has emerged as a fruitful strategy for executing drug candidates, so little attention has been given to their potential toxic side effects and the interaction potentials of these bioactive tripeptides with DNA, RNA, other proteins, or other subcellular structures were neglected. In this context, the investigation of cytologic and genetic damage potentials of novel GPEs such as GPE1, GPE2, and GPE3 is literally worthwhile for developing further and may contribute to the generation of novel disease-modifying agents [6]. Based on these earlier findings, the main objective of the current study was to investigate the cytotoxic, oxidative, and genotoxic damage potentials of novel GPE analogs in comparison to natural GPE in peripheral human whole blood (PHWB) cells for the first time. To further evaluate the toxicity potentials of GPE analogs, these three novel GPEs were also assessed for their embryotoxic effects on cultured human pluripotent NT2 embryonal carcinoma cells. We assessed the *in vitro* effects of these peptidomimetics on cell viability using MTT and LDH release assays, antioxidant capacity using TAC assays, and DNA damage response using SCE, MN, and 8-OH-dG assays. In addition, NDI analysis was performed on cytostatic action potentials by novel GPEs.

## 2. Materials and Methods

Synthesis of GPE and GPE1-3 was performed as reported by Marinelli et al. [5]. All reagents for the synthesis of compounds were purchased by Sigma–Aldrich, MO, USA).

### 2.1. Experimental Design

For assessing the biosafety of novel tripeptides, human peripheral blood samples were used. The cell cultures were designed due to minor modifications of the previously recommended protocol [16]. Heparinized blood samples were obtained from five healthy male volunteers, nonsmoking and nonalcoholic and with no recent history of exposure to mutagens; aged 24–28 years (26.4 ± 1.8). The study was approved by the local Ethics Committee (2019/03–30) and was in accordance with the Declaration of Helsinki and the International Conference on Harmonization for Good Clinical Practice. Written informed consent was obtained from all patients. The 0.6 ml of blood was cultured in 6.4 ml of culture medium (Karyotyping Medium, Gibco, MA, USA) with 5.0 mg/ml of phytohemagglutinin (Sigma–Aldrich, USA). The GPE and novel GPEs were added into the culture tubes at six different concentrations (0.1, 1, 10, 25, 50, and 100 *μ*M) just before the incubation for 72 h.

### 2.2. Cytotoxicity Testing

Cell viability was determined using MTT and LDH release assays. The cell proliferation rate was determined by using commercially available MTT kits (Cayman Chemical Company, USA). Triton-X (%1, Sigma-Aldrich) was used as a positive control in cytotoxicity testing. In brief, cells were incubated with GPEs at 37°C for 72 h. MTT agent was added into the cultures for 3.5 h and generated formazan crystals were dissolved in dimethyl sulfoxide (Sigma-Aldrich). Finally, absorbances of samples in the well plates were read at 570 nm by an ELISA plate reader (Bio-Tek, Winooski, VT, USA) [17].

LDH release assay was carried out by using the commercially available LDH-cytotoxicity assay kit (Cayman Chemical, USA) due to the provider's guide. In brief, after the 72 h incubation period, the obtained 100 *μ*L supernatant was transferred to a fresh well of 96-well plate containing 100 *μ*L of the reaction mixture from the kit and incubated for 30 min at room temperature. After this extra incubation, the absorbances of the sample were read at 490 nm using a microplate reader (Bio-Tek). Finally, the total amount of released LDH was calculated using the equation % LDH released = (LDH amount in medium/total LDH amount) × 100 [13, 18].

### 2.3. TAC Analysis

Plasma samples, obtained by centrifugation of the whole blood cultures at 2000 g for 10 min 72 h after incubation with GPEs, were analyzed using commercially available TAC assay kits (Rel Assay Diagnostics, Gaziantep, Turkey). Ascorbic acid (C_6_H_8_O_6_, Sigma-Aldrich, 20 *μ*M) was used as the positive control group [19].

### 2.4. Genotoxicity Testing

For scoring SCE formations, 5-bromo-2-deoxyuridine (BrdU, Sigma-Aldrich) was added at the initiation step. Exactly 70 h and 45 min after the beginning of the incubations, demecolcine (N-Diacetyl-Nmethylcolchicine, Sigma-Aldrich) was added into the culture tubes. After hypotonic treatment (0.075 M·KCl), three repetitive cycles of fixation with methanol/acetic acid solution (3 : 1, v/v), centrifugation, and resuspension steps, the suspension was dropped onto microscopic slides, following differentially stained for examination of the SCE rates according to the fluorescence plus Giemsa (FPG) procedure. For each application, thirty metaphases (containing 42–46 chromosomes per cell) were scored and the values obtained were presented as SCEs per cell [20, 21]. A negative control and a positive control (mitomycin-C (MMC), Sigma, 10^−7^ M) were used to ensure the validity of the genotoxicity testing.

The MN test was implemented by adding cytochalasin B (Sigma) after 44 h of culture initiation. At the end of the 72 h incubation period, the lymphocytes were fixed with ice-cold methanol/acetic acid (1 : 1, v/v). The fixed cells were transferred to microscopic slides and then stained with Giemsa solution (Sigma). The criteria for scoring MN were applied according to the previous protocol [22]. At least 2.000 binucleated cells were examined per treatment for the presence of one, two, or more MN. Cytostatic potentials of GPEs were also assessed by the nuclear division index (NDI). To determine NDI rates, 500 cells per application were examined for the presence of one, two, or more than two nuclei, and the nuclear division index (NDI) was determined as

(1) $$\begin{array}{c} \text{NDI}=\frac{\left[1N+\left(2\times 2N\right)+\left(4\times >2N\right)\right]}{C}\text{,} \end{array}$$

where 1*N* represents the number of cells with one nucleus, 2*N* with two nuclei, and >2*N* with more than two nuclei, and *C* represents the number of scored cells [23].

8-hydroxy-2′-deoxyguanosine assay kits were provided from Cayman Chemical® for measuring 8-OH-dG levels *in vitro* due to the provider's guide [24, 25].

### 2.5. Embryotoxicity Testing

The human pluripotent embryonal carcinoma NT2 cells were obtained from the American Type Culture Collection (ATCC). NT2 cells were cultured in RPMI-1640 medium (at 37°C, in 5% CO_2_) containing 10% FBS, 100 *μ*g/ml of streptomycin, and 100 U/ml of penicillin (Thermo Fisher, USA). GPEs at their multiplexed concentrations (0.1, 1, 10, 25, 50, and 100 *μ*M) were added to the well plates and cultured for 72 h (*n* = 5). Embryotoxicity potential of GPEs was evaluated using MTT and LDH release assays. Cyclophosphamide (CPA, 10 *μ*g/ml, Sigma–Aldrich, USA) was used as a positive control.

### 2.6. Statistical Analysis

The obtained data are presented by mean ± SD. from five independent repetitions. For statistical evaluation, one-way analysis of variance (ANOVA) and Duncan's test were performed using the statistical program SPSS version 20.0. Statistical decisions were made with a significance level of 0.05.

## 3. Results

**Figure 2:**
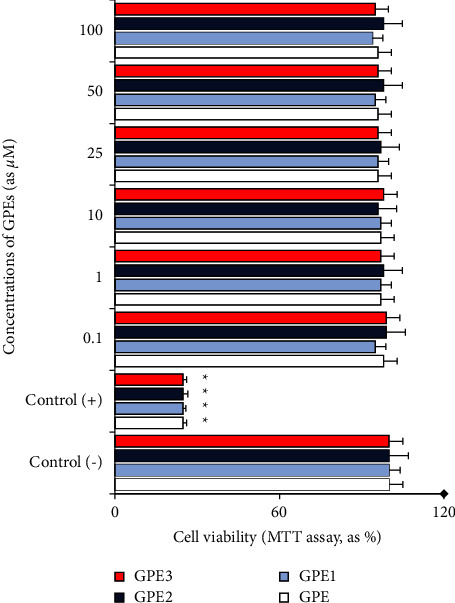
The rates of PHWB cell viability after 72 h exposure to different concentrations of GPEs. The results were presented as a percentage of the control group (*n* = 5). Data are expressed as mean ± SD *p* < 0.05^*∗*^.

**Figure 3:**
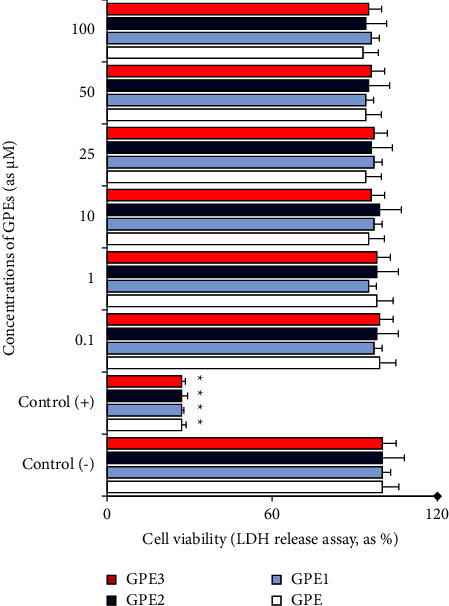
Extracellular LDH levels in cultured PHWB cells treated with different concentrations of GPEs *p* < 0.05^*∗*^.

The MTT and LDH release assays were applied to determine the cytotoxic response to different concentrations of GPEs (0.1 to 100 *μ*M). The positive control (as %1 Triton-X) caused significant (*p* < 0.05) decreases of cell viability in comparison to the untreated (negative control) group at rates of 75.14% and 72.89, in MTT and LDH release assays, respectively. By contrast with this status, when the cultured PHWB cells were exposed to all GPEs, the observed cell viability rates were not statistically different from the untreated cultures (Figures 2 and 3). The cytotoxicity testing determined the noncytotoxic feature of GPE and its novel analogs.

We determined the TAC levels after application with GPE and GPE analogs. The results of the TAC assay revealed that all examined tripeptides led to statistically significant (*p* < 0.05) elevations of antioxidant capacity levels as compared to untreated PHWB cells. The established decreasing order of effectiveness of all tested tripeptides in elevating antioxidant capacity was GPE3 > GPE > GPE2 > GPE1 (Table 1).

**Figure 4:**
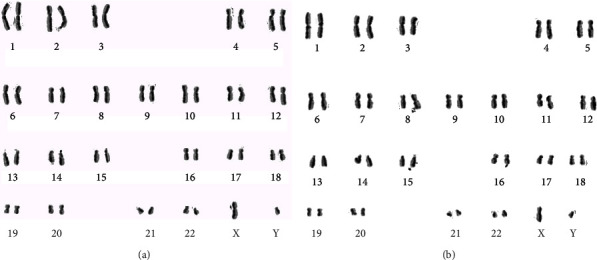
Sample SCE metaphases: (a) control (+) as MMC; (b) GPE3 (100 *μ*M) treated cultures.

**Figure 5:**
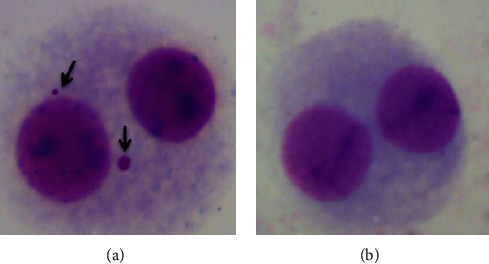
Sample binucleated cells: (a) control (+) as MMC; (b) GPE3 (100 *μ*M) treated cultures (arrows show MN formations).

The results of the SCE testing were shown in Table 2 and Figure 4. The SCE frequencies of the cells treated with six different concentrations (0.1, 1, 10, 25, 50, and 100 *μ*M) of GPEs were not statistically different from negative control values. The SCE frequencies per cell significantly increased in human lymphocyte cultures treated with MMC as positive controls relative to the negative control (*p* < 0.05). Likewise, the results of the MN experiments with GPEs are also summarized in Table 2. No evidence of marked clastogenicity and aneugenic effect were observed following 72 h treatments with GPEs (Figure 5). Thus, it is concluded that treatments with GPEs did not induce SCE or MN formations in cultured human peripheral blood lymphocytes.

As shown in Table 2, MMC led to a statistically significant reduction in the NDI as compared to the control groups. But GPEs at all tested concentrations did not decrease the NDI to the same extent as the negative control. Therefore, GPEs were concluded as noncytostatic even at the highest concentration (100 *μ*M) for the 72 h treatment period.

**Figure 6:**
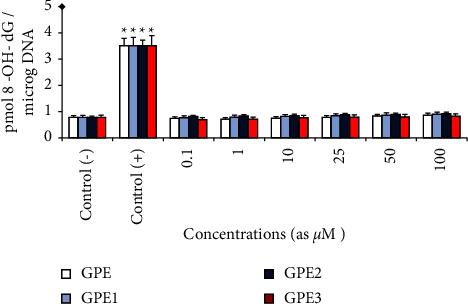
The levels of 8-oxo-2-deoxyguanosine (8-OH-dG) adduct levels in human blood cultures treated with GPEs for 72 h *p* < 0.05^*∗*^.

The level of 8-OH-dG in cultured human blood cells of the control and GPE, GPE1, GPE2, and GPE3 treated groups is shown in Figure 6. The level of 8-OH-dG, which is a sensitive marker of oxidative DNA damage, was elevated after exposure to MMC. In fact, it was determined that MMC (at 10^−7^M) significantly elevated 8-OH-dG levels (about 4.5 folds) in human blood cultures after 72 h. Contrariwise, 8-OH-dG levels were not increased in human blood cell cultures that were applied with all GPEs at different concentrations ranging from 0.1 to 100 *μ*M.

After treatment with CPA, this agent induced significant decreases in cell viability rates in NT2 cells as compared to untreated cells. In fact, 10 *μ*g/ml of CPA induced cell death at rates of 82.55% and 86.80%, in MTT and LDH release assays, respectively. On the contrary, all tested concentrations of GPEs did not alter the cell viability rates in comparison to untreated cell cultures. Hence, GPEs were concluded to exert nonembryotoxic action in *in vitro* conditions.

## 4. Discussion

The present results from cytotoxicity and genotoxicity testing clearly revealed that GPEs had good biosafety profiles and were trouble-free from the toxicological point of view. As a matter of fact, in this study, all treatments with GPE and GPE analogs did not change the viability (by MTT and LDH release assays) of cultured human blood cells as compared to untreated cultures. In accordance with our findings, the GPE did not alter the cell viability rates detected using ELISA and autoradiography methods in cultured rat hippocampal neurons [4, 26]. Likewise, the performed MTT and LDH release assays propounded noncytotoxic properties of both lipoic acid conjugated GPE (LA-GPE) and natural GPE on differentiated human SH-SY5Y neuroblastoma cell cultures [6]. Moreover, treatment with GPE (100 *μ*M) elevated the proliferation rates of mouse embryonic neural stem cells [27].

In addition to the results of these *in vitro* studies, a previous *in vivo* investigation on the cortex and hippocampus regions of experimental rats revealed the neuroprotective action of natural GPE. The cytoprotective action by GPE was attributed to an associated mechanism for central and systemic antiapoptotic effects of IGF-1 [28]. The exact molecular mechanisms underlying the noncytotoxic and cytoprotective actions of GPE on human blood cells are still unclear. However, GPE was shown to inhibit apoptosis and promote cell survival in human corneal fibroblast cultures via stimulating a signaling pathway through phosphatidylinositol (PI) 3-kinase and then protein kinase B also known as Akt [29]. It was reported that activated Akt modulates fundamental cellular events involving cell proliferation and survival via phosphorylating variegated substrates [30]. Our results also revealed that not only GPE but also GPE1, GPE2, and GPE3 were noncytotoxic in human blood cells. The observed feature of the novel GPE analogs might be associated with the activation of the PI3K/Akt pathway. In fact, PI3K/Akt pathway activation inhibited apoptosis of chicken splenic lymphocytes *in vitro* and stimulated their proliferation [31].

Systemic administration of GPE was found to activate nuclear erythroid 2-related factor 2 (Nrf2), a transcription factor-induced antioxidant response in Parkinson's model-generated animals [32]. Likewise, the neuroprotection by LA-GPE and GPE was reported to be related to the expression of antioxidant proteins in differentiated human SH-SY5Y cells [12]. In supporting the presence of antioxidant properties by GPE, the present results proved that GPE along with its three analogs exhibited a high level of antioxidative action on human blood cells. Furthermore, the present findings also indicated that GPE3 exhibited higher potency in supporting antioxidant capacity than GPE in human blood cells. The differences in antioxidant potentials of GPEs were highly contingent upon the sequence and composition of amino acids. Indeed, Gly residue makes a major contribution to antioxidant capacity because of that Gly is a proton-donating source and may combat free radicals. In addition, the single hydrogen atom of Gly may positively influence the antioxidant capacity. Similar to Gly residue, the beneficial role of Pro residue in advocating antioxidant activity was previously put forward on peptide purified from protein hydrolyzate of *Saccharomyces cerevisiae* [7, 33–35].

In a previous report, it was reported that cytoprotective action by Nrf2 was glutathione-dependent. And the increased cell death rates were directly associated with elevations in the amounts of reactive oxygen and nitrogen radicals that entailed DNA damage [36]. GPE, GPE1, GPE2, and GPE3 were found to be nongenotoxic (by SCE, MN, and 8-OH-dG testing) and noncytostatic (by NDI analysis) in human lymphocytes in the present study. At this point, the determination of cyto- and geno-toxicological profiles is of critical importance for the use of GPE and its novel analogs, most particularly GPE3 as drug candidates. Nominately it was previously identified that eptastigmine readily entered into the CNS and suppressed acetylcholinesterase for a prolonged period. But its observed haemato-toxic effects have intervened in eptastigmine usage in further clinical trials against Alzheimer' Disease [37, 38].

A few investigations were undertaken to reveal the teratogenic and embryotoxic action potentials by different neuropeptides like L-prolyl-L-leucyl-glycinamide, cyclo(glycyl-L-leucyl)[Cyclo(Gly-Leu)] and cyclo(1-aminocyclo-pentanecarbonyl-L-alanyl) (cyclo(Acp-Ala)) and found positive results on *in vivo* models [39]. Interestingly, some antioxidant-featured amino acid derivatives L-methionine- or N-acetylcysteine-led to developmental toxicity in sea urchin embryos and gametes [40]. On the contrary, our *in vitro* results revealed that tested GPE and their GPE analogs exerted nonembryotoxic features. In this regard, the embryotoxicity or teratogenicity evaluation of peptide-based drug and drug candidates is critically important in the drug development process. So, it was reported that about 5% of the pregnancies used potential embryotoxic or teratogenic drugs during pregnancy and 0.66% of them were in the risk category [41]. Our findings suggest that GPEs do not exert potentially embryotoxic action for impairing the pluripotent lineage of NT2 cells, and these data will contribute to gain new embryotoxicity insights into their potential for safe use during pregnancy.

In a conclusion, proven noncytotoxic, nongenotoxic, noncytostatic and nonembryotoxic features of GPE analogs are worthwhile exploring further and have high potentials for improving the development of novel disease-modifying agents in the treatment of variegated disorders.  Table 3.

## Figures and Tables

**Table 1:** The effects of GPE and novel GPE analogs on TAC level (as mmol Trolox Equiv./L) in cultured PHWB cells. Different letters indicate statistically significant differences (*p* ≤ 0.05).

Groups	TAC level
Untreated cells	4.22 ± 0.78^a^
Ascorbic acid	17.25 ± 2.32^e^

GPE	
0.1 *μ*M	4.41 ± 0.53^a^
1 *μ*M	5.09 ± 0.51^a^
10 *μ*M	7.66 ± 0.81^b^
25 *μ*M	9.14 ± 1.14^c^
50 *μ*M	10.06 ± 1.08^c^
100 *μ*M	11.91 ± 1.37^c^

GPE1	
0.1 *μ*M	4.12 ± 0.50^a^
1 *μ*M	4.35 ± 0.58^a^
10 *μ*M	4.86 ± 0.47^a^
25 *μ*M	6.14 ± 0.62^b^
50 *μ*M	7.90 ± 0.74^c^
100 *μ*M	10.45 ± 1.11^c^

GPE2	
0.1 *μ*M	4.12 ± 0.43^a^
1 *μ*M	4.86 ± 0.54^a^
10 *μ*M	6.77 ± 0.61^bc^
25 *μ*M	9.18 ± 1.08^c^
50 *μ*M	10.90 ± 1.25^c^
100 *μ*M	11.75 ± 1.41^c^

GPE3	
0.1 *μ*M	4.96 ± 0.38^a^
1 *μ*M	5.77 ± 0.51^ab^
10 *μ*M	8.52 ± 0.79^c^
25 *μ*M	10.55 ± 1.06^c^
50 *μ*M	14.30 ± 2.43^d^
100 *μ*M	16.78 ± 2.29^e^

**Table 2:** The effects of GPEs on SCE and MN rates in cultured human lymphocytes. ^*∗*^symbol presents a statistical difference from the control (-) group at the level of *p* < 0.05.

Groups	SCEs/cell	MN/2000 cells	Nuclear division index (NDI)
Untreated cells	4.55 ± 0.78	0.91 ± 0.15	1.37 ± 0.26
MMC	10.92 ± 1.06^*∗*^	3.74 ± 0.52^*∗*^	1.09 ± 0.21^*∗*^

GPE			
0.1 *μ*M	4.16 ± 0.61	0.79 ± 0.18	1.38 ± 0.22
1 *μ*M	4.06 ± 0.59	0.83 ± 0.22	1.42 ± 0.14
10 *μ*M	4.38 ± 0.41	0.88 ± 0.15	1.39 ± 0.31
25 *μ*M	4.46 ± 0.73	0.85 ± 0.19	1.36 ± 0.25
50 *μ*M	4.41 ± 0.36	0.89 ± 0.20	1.34 ± 0.24
100 *μ*M	4.47 ± 0.63	0.90 ± 0.18	1.32 ± 0.21

GPE1			
0.1 *μ*M	4.29 ± 0.43	0.81 ± 0.15	1.44 ± 0.29
1 *μ*M	4.47 ± 0.55	0.84 ± 0.23	1.41 ± 0.33
10 *μ*M	4.55 ± 0.69	0.90 ± 0.22	1.37 ± 0.23
25 *μ*M	4.52 ± 0.56	0.94 ± 0.17	1.34 ± 0.28
50 *μ*M	4.63 ± 0.48	0.98 ± 0.21	1.32 ± 0.22
100 *μ*M	4.65 ± 0.53	1.02 ± 0.24	1.42 ± 0.24

GPE2			
0.1 *μ*M	4.38 ± 0.36	0.85 ± 0.13	1.38 ± 0.30
1 *μ*M	4.51 ± 0.50	0.89 ± 0.19	1.37 ± 0.25
10 *μ*M	4.58 ± 0.47	0.91 ± 0.16	1.34 ± 0.27
25 *μ*M	4.66 ± 0.60	0.92 ± 0.26	1.30 ± 0.33
50 *μ*M	4.69 ± 0.51	1.01 ± 0.23	1.28 ± 0.27
100 *μ*M	4.71 ± 0.66	1.04 ± 0.20	1.22 ± 0.24

GPE3			
0.1 *μ*M	4.18 ± 0.44	0.77 ± 0.17	1.45 ± 0.29
1 *μ*M	4.29 ± 0.51	0.76 ± 0.21	1.43 ± 0.26
10 *μ*M	4.23 ± 0.40	0.82 ± 0.24	1.41 ± 0.31
25 *μ*M	4.35 ± 0.36	0.85 ± 0.16	1.42 ± 0.34
50 *μ*M	4.45 ± 0.56	0.88 ± 0.28	1.40 ± 0.27
100 Μm	4.61 ± 0.47	0.92 ± 0.23	1.38 ± 0.25

**Table 3:** The embryotoxic action potentials by GPEs on cell viabilities in cultured human pluripotent embryonal carcinoma NT2 cells. ^*∗*^symbol presents a statistical difference from the control (-) group at the level of *p* < 0.05.

Groups	Concentrations	Cell viability (%, MTT)	Cell viability (%, LDH release)
Untreated cells		100	100
CPA		17.45 ± 3.28	13.20 ± 2.85
GPE	0.1 *μ*M	98.60 ± 5.54	98.24 ± 7.12
	1 *μ*M	97.40 ± 4.66	98.45 ± 6.54
	10 *μ*M	97.14 ± 6.96	96.20 ± 5.87
	25 *μ*M	98.10 ± 5.80	97.12 ± 7.91
	50 *μ*M	95.42 ± 6.62	94.22 ± 6.97
	100 *μ*M	93.78 ± 6.44	92.95 ± 8.24

GPE1	0.1 *μ*M	97.48 ± 6.22	98.33 ± 4.33
	1 *μ*M	98.11 ± 6.87	97.14 ± 5.18
	10 *μ*M	94.35 ± 8.12	96.44 ± 5.23
	25 *μ*M	92.46 ± 7.33	95.10 ± 5.88
	50 *μ*M	90.81 ± 5.19	95.35 ± 6.13
	100 *μ*M	89.50 ± 8.77	91.75 ± 6.34

GPE2	0.1 *μ*M	99.30 ± 5.33	97.75 ± 7.42
	1 *μ*M	98.25 ± 5.97	98.06 ± 6.90
	10 *μ*M	97.65 ± 7.66	97.22 ± 8.12
	25 *μ*M	97.21 ± 6.82	96.19 ± 7.41
	50 *μ*M	94.22 ± 7.16	94.50 ± 7.79
	100 *μ*M	91.56 ± 7.54	92.80 ± 7.72

GPE3	0.1 *μ*M	98.44 ± 7.10	99.11 ± 7.89
	1 *μ*M	99.26 ± 7.61	98.46 ± 8.41
	10 *μ*M	97.43 ± 6.90	98.72 ± 8.33
	25 *μ*M	94.82 ± 6.75	96.80 ± 7.15
	50 *μ*M	92.75 ± 7.32	95.52 ± 6.02
	100 *μ*M	93.35 ± 6.80	92.68 ± 5.90

## Data Availability

The authors confirm that the data supporting the findings of this study are available within the article.
